# Decision-making and acute behavioural disturbance (ABD): a qualitative thematic analysis of perspectives on decision-making by UK ambulance paramedics

**DOI:** 10.1186/s12873-025-01297-7

**Published:** 2025-07-26

**Authors:** Jaqualine Lindridge, Timothy Edwards, Leda Blackwood

**Affiliations:** 1 Department for Health, University of Bath, Bath, Somerset England; 2 London Ambulance Service NHS Trust, London, England; 3 Department of Psychology, University of Bath, Bath, Somerset England; 4 South East Coast Ambulance Service NHS Foundation Trust, Crawley, West Sussex England

**Keywords:** Acute behavioural disturbance, Agitation, Excited delirium, Paramedic, Decision-making, Restraint, Emergency medical services

## Abstract

**Background:**

Incidents involving severe agitation are complex emergencies which occur infrequently in the community but have high stakes for patients and responders. Acute behavioural disturbance (ABD), an umbrella term used to describe severe agitation, sometimes known as excited delirium, affects patients who typically present to police and ambulance paramedics and may require restraint. However, little is known about how paramedics make these restraint decisions. This research aimed to explore the decisions made by paramedics when managing restraint in the context of ABD in the UK ambulance setting.

**Methods:**

Ten semi-structured interviews and one focus group were undertaken with one newly qualified and 12 experienced paramedics employed by a large metropolitan ambulance service. The resulting data were analysed using reflexive thematic analysis, informed by critical realism.

**Results:**

We identified three interrelated themes from the data: working in the context of fear, navigating complexity without consistent and adequate formal subject-specific training, and the nature of professional roles and relationships at incident scenes. These restraint decisions are a source of concern, with paramedics fearing patients coming to harm and the potential for detrimental professional repercussions. Decision-making was complicated by mismatched levels of education specific to restraint and the management of ABD patients, and the requirements of ‘real world’ clinical practice. The social relationships between paramedics and others at incident scenes were also important, with paramedics situating ABD incidents as healthcare rather than law enforcement encounters key to influencing restraint decisions.

**Conclusions:**

Our results indicated that paramedics would benefit from further education which is specific to identifying and managing the complex social interactions which occur at ABD scenes. High-fidelity simulation training delivered jointly with other agencies may be a helpful intervention in achieving this.

**Supplementary Information:**

The online version contains supplementary material available at 10.1186/s12873-025-01297-7.

## Background

Acute behavioural disturbance (ABD) refers to a severe presentation of agitation which has been linked to several deaths in the context of physical restraint delivered by law enforcement personnel [1, 2] and pharmacological restraint provided by paramedics [3]. In this exploratory, qualitative study, we examine paramedics’ decision-making when at ABD incidents in the UK pre-hospital ambulance setting where patients may require physical and/ or pharmacological restraint. Our research seeks to gain a deeper understanding of decision-making in this clinical scenario, to inform developments in this area of practice. Firstly, we will provide some background and context to this topic, before reviewing the literature in two contexts: how paramedics make decision in relation to restraint, and how paramedics make decisions in general.

ABD is an umbrella term used to describe significant agitation or disordered behaviour, with or without physiological compromise [4]. Whilst a recent Delphi study concluding that the term should be replaced with ‘red flag agitation’ [5] it is widely used in the UK [4, 6, 7]. Other terms are used internationally, including excited delirium syndrome [8] and excited delirium [9]. The features listed in clinical guidance, such as superhuman strength and imperviousness to pain have been said to invite forceful responses [10]. Much concern has been expressed that the term excited delirium when used in relation to cause of death diverts attention from the causal role of physical restraint in these deaths and serves to erode police accountability for deaths in custody [11–13]. It has also been suggested that use of the term ‘excited delirium’ dehumanises agitated people, and infers that violence and restraint is inevitable [14, 5]. Acknowledging this controversy, the term ABD is used throughout this study because it was the term in predominant use in the UK at the time of the study, and that most familiar to the study participants.

The definition of both a ‘paramedic’ and an ‘ambulance crew’ varies internationally. The required qualifications, scope of practice, and requirements for registration with a statutory regulatory body varies from country to country. In some emergency medical systems, ambulances are staffed by registered nurses (e.g. Sweden [15]), in others registered paramedics (e.g. Australia [16] and UK [17]), and in others by paramedics and emergency medical technician (EMTs) who are certified and/ or licensed to practice (e.g. the United States [18]).

UK paramedics are regulated healthcare professionals registered with the Health and Care Professions Council (HCPC), a statutory body which regulates paramedics and other healthcare professionals in the UK. The title ‘paramedic’ is legally protected, and they currently require a bachelor’s degree to gain entry onto the professional register [19] and work autonomously within a defined scope of practice, which includes the administration of specific medicines according to schedules 17 and 19 of the Human Medicines Regulations 2012 [20]. This research was conducted at a large metropolitan ambulance service which provides free at-the-point-of-access emergency medical services. A typical response to a high-acuity emergency at the study site is the attendance of an ambulance, staffed by a paramedic and a non-registered healthcare worker such as an EMT [17]. A well-established critical care advanced practice service is in operation there and deploys advanced paramedics (APs) to suspected cases of ABD, who in addition to the standard formulary for paramedics are permitted to administer sedation using Patient Group Directions (PGDs), a legal framework which enables the administration of parenteral medicines without a prescription [21].

We undertook a literature review to identify studies on decision-making in two contexts. Firstly, the restraint of patients experiencing ABD (or similar presentation of agitation) in any clinical practice setting or by any healthcare professional group, and secondly, decision-making by paramedics in general.

A scoping review of patient safety in ambulance services identified a relative lack of literature about restraint in the ambulance setting [22]. Recent Norwegian studies found that physical coercion was influenced by the need to protect both patients and professionals from harm, and that ambulance personnel made decisions in the context of fear and insecurity in relation to how much post-event support they could expect from their employers [23], and the degree to which they were explicitly permitted to use force in practice [24]. Elsewhere, it has been reported that nurses consider restraint to be a necessary, but undesirable activity which contradicts their caring role [25–27], and research in the in-patient psychiatric setting highlights a reliance on tacit knowledge when making restraint decisions [25]. However, no literature was found that touches on restraint decision-making by paramedics specifically in the context of ABD.

Turning to clinical decision-making more broadly, some studies suggest the complexity of decisions in paramedicine exceeds what is accounted for in guidelines [28, 29]. A mismatch between information need and information availability has also been identified as a barrier to decision-making in cardiac arrest management [30] and end-of-life care [31]. Other research has identified that paramedics do not feel sufficiently prepared by their formal training to manage behavioural and mental health emergencies and rely on tacit knowledge and experience [32, 33].

The literature also reveals that paramedics fear blame and repercussions such as disciplinary investigation and litigation in several areas of clinical practice including managing patients who self-harm [32]; making decisions about the discharge of care, the need for hospital admission and transfer [29, 34–36]; and cardiac arrest management [37, 38]. There are also concerns about a lack of support from employers concerning making discharge of care decisions [29, 39].

Paramedic decision-making is currently thought to be based on atomised individual-level ‘rational’ processes, with studies focusing on cognitive processing [40–42]. Decisions about restraint however take place in a social context which includes other professionals, such as the police and other healthcare workers, non-paramedic ambulance workers (in the UK context this includes non-registered ambulance clinicians such as EMTs and Associate Ambulance Practitioners) and bystanders. This context is important. In their study of cardiac arrest decision-making, Brandling et al. observed the influence of police officers on clinical decisions [38], with ambulance clinicians (including paramedics) reporting difficulty in challenging police decisions about resuscitation. Other studies have acknowledged the pressure felt from bystanders to make particular treatment decisions; such as pharmacological interventions in seizure management [34] or whether to convey patients to a hospital when at the end of their lives [31].

Our literature review suggests decisions may be complex due to factors such as risk tolerance, training, and prognostic uncertainty [28, 29, 37], and understanding this complexity may improve the provision of quality care. Whilst there has been some relevant research undertaken about other professions [26], restraint-related research is limited in relation to the paramedic profession. This study explored the decisions made by paramedics when managing a restraint incident in the context of ABD and sought to identify opportunities to improve practice.

## Methods

### Research philosophy and methodology

Our research was conducted from a critical realist perspective, following Campbell [43] and Maxwell’s [44] approach which combines ontological realism and epistemological constructivism. Ontological realists consider that reality, and the constituents of reality, exist independently of consciousness [45], and epistemological constructivists consider that our understanding of reality is constructed based on human perception [44]. Our position is that the phenomenon of decision-making in the context of ABD is real and that paramedics make sense of this in an individual and socially constructed manner.

Braun and Clarke’s [46] method of reflexive thematic analysis (RTA) was used to analyse the data. RTA recognises and values the subjectivity of the researcher, which is consistent with our critical realist epistemology. It is a theoretically flexible analytic method which involves the systematic coding of data and the identification and interpretation of patterns within the data. Data analysis began following the first interview and followed an exploratory and iterative approach [47].

### Recruitment

The study was advertised using internal bulletins, social media, and word of mouth. Thirteen participants were recruited between May and November 2021. Participants were purposively sampled from the generalist paramedic population to provide a balanced sample in relation to gender and type of pre-registration training. Paramedics with additional or specialist post-registration education and/ or skills in managing acutely agitated patients were not included in this sample group, such as APs in critical care. This is because their views and experiences were anticipated to be different from generalist paramedics, due to this and increased exposure to ABD patients. A connected study sampling APs and reporting their perspectives has been recently published [48].

There is no agreement on the sample size required for qualitative studies [49]. We followed Braun and Clarke’s recommendation that researchers “…*make an in-situ decision about the final sample size*,* shaped by adequacy (richness*,* complexity) of the data for addressing the research question…”* [50]. Our research focussed on a specific geographical area and all participants were able to discuss several cases from their personal experiences in depth, and good quality dialogue was produced as a result.

### Participant demographics

Of the 13 participants, one participant was a newly qualified paramedic (NQP) and within their two-year preceptorship period. The remaining 12 participants were experienced paramedics, with a minimum of two years of post-qualifying clinical experience. Five participants were female and eight were male which broadly aligns with the gender balance of paramedics in the UK at the time [51]. There was variation in the level of participant’s paramedic qualifications, which are outlined below (Table 1), reflecting historical variation in pre-registration requirements.

**Table 1:** Educational levels of participants

Educational Level	Number of Participants
Bachelor’s degrees in paramedic science/ paramedicine (FHEQ L6)	8
Certificate of higher education, or equivalent (FHEQ L4)	3
Foundation degree (FHEQ L5)	1
Post-graduate qualification (FHEQ L7).	1

Five participants undertook their qualifying education in either Australia or New Zealand with the remaining participants qualifying in the UK. At the time of the study approximately 25% of the study site’s paramedic workforce were trained in Australia [52].

### Procedure

Ten semi-structured, in-depth interviews lasting between 53 and 64 min, and one group interview with three participants lasting 106 min were undertaken between May and November 2021. Interview guides are available online as supplementary material. Participants were asked to tell stories about ABD incidents they had attended, and probed on how and why they made the decisions they did. Interviews were conducted with the least interviewer input as possible to allow participants to talk about what was meaningful for them [53]. All participants spoke freely and in depth about their experiences. As a paramedic themselves, the interviewer was an insider researcher and shared professional experiences, identity and language with participants [54]. Insider research benefits from early acceptance of the interviewer and can facilitate openness and access to data which can be difficult to obtain from outsider research [55, 56]. There is a risk in insider research for interviewees to assume likeness with researchers and constrain their explanations as a result, and for researchers to confuse their observations with those of participants [55]. To mitigate this, informal member checking took place during interviews with the facilitator summarising data and then questioning participants to determine accuracy.

Data collection took place during the COVID-19 pandemic and interviews were conducted and digitally recorded using Microsoft Teams™, before being transcribed intelligent verbatim. Data were anonymised during transcription, and participants were assigned pseudonym identifiers. In total, the individual and group interviews generated 360 pages of data, consisting of 99,011 words. Interviews produced rich and complex data with a high level of depth. Based on the quality of the interview dialogue, these interviews were deemed to have generated sufficient relevant data to enable the development of meaningful themes.

### Analysis

Six phases of thematic analysis were used to structure the data analysis [57]. Firstly, the first author (JL) became familiar with the dataset through listening to the recordings, transcribing, reading and re-reading the data. In the second stage JL systematically identified extracts of data which were relevant to the research question and applied initial latent and semantic codes, which were worked into candidate themes in the next stage using a diagramming approach to identify patterns in the data. In the fourth stage, all three co-authors (JL, TE and LB) discussed the candidate themes in relation to the data and study aims and revised the initial theme categories. These were then further refined in the fifth phase and finally written up by weaving together analytic narrative and data extracts.

Throughout each stage, the first author made memos noting their reflections and interpretations of the data, noting their context and view of the topic. As an insider researcher, the first author considered their assumptions and perspectives about the data. The data were frequently discussed by the co-authors during phases four to six, where the findings were reviewed and debated. Ethical approval for this study was provided by the West Midlands – Black Country Research Ethics Committee (REC Reference 21/WM/0028), Heath Research Authority (Project ID: 265010).

## Results

We identified three themes (Table 2).

**Table 2:** Theme summaries

Theme	Summary	Example data extract
Working in the context of fear	Fears of patients coming to physical or mental harm as a result of physical restraint, a fear of things going wrong, and adverse consequences for the paramedics themselves	*“… it’s not a comfortable position to be in because we don’t know what we’re doing*,* and we know that it’s incredibly high risk.”*. - H10
Navigating complexity without adequate formal subject-specific training	A perceived lack of pre-registration training and education in ABD about both clinical and medico-legal issues with knowledge acquisition occurring by chance, a mismatch between training and the realities of ‘real-world’ clinical practice, and a lack of clarity about the boundaries of paramedic autonomy and scope of practice.	“*Probably one of the most high-risk jobs physically for us and the patient*,* that we come with the least equipment and training for*.” - H8
Nature of professional roles and relationships at incident scenes	Dealing with issues related to whether restraint was understood as within the remit of paramedics and whether ABD incidents were understood as being medical or law enforcement events	‘*some police officers may see […] the decision to restrain as their decision […] actually*,* as a health care professional […] I have clinical responsibility for this patient.*’ – H11

### Theme 1: Working in the context of fear

In this first extract, H5, notes that they are untrained in physical restraint and describes when they would consider undertaking it.

*I don’t know how to restrain anyone. That idea scares me. I will try and avoid that at all costs and I think the only time I would ever try it is if […] it’s their life on the line. – H5*

H5 is fearful of being in a position of having to provide physical restraint. Recognising their lack of formal training this is an action of absolute last resort, to be avoided and only considered where not doing so would lead to far greater harm. H5 sheds light on the challenges which paramedics experience when in situations for which they have limited education and training, but are required to manage none-the-less.

In the next extract, H1, an overseas-trained paramedic reflects on UK paramedics’ concerns about making decisions about patients’ capacity to make decisions.

*People seem very scared of the laws, of the capacity laws, the mental health laws and seem very worried they will overuse capacity laws and overly deprive someone, whereas really they are having a very serious medical event which needs treating. - H1*

In comparing paramedics’ fears of ‘overly’ depriving a person of their liberty to the severity of the patient’s ‘*very serious*’ clinical condition and need for care, H1 illuminates an important tension for paramedics in prioritising meeting patients’ clinical needs within the restrictions of a legal framework designed to protect patient autonomy, and their fears of repercussions should they get this wrong. There is a risk here that the fear of repercussions will inhibit the decision to intervene and provide clinical care.

In the next example, H9, another overseas-trained paramedic recalled the cautionary words of tutors during their UK conversion training.

*The words ‘watch yourself’ came up quite a lot during my training. Just make sure that you are doing whatever is legal or necessary or justifiable. I can’t remember ever being told of what protections I’ve got. – H9*

H9’s comments here reveal a defensive tone to their training, with a lack of explicit instruction. Their training was underlined with an inference that there is some personal risk to the practitioner associated with these decisions. In remembering being told to ‘watch themselves’, H9 brings insight into the perceived riskiness of restraint for paramedics. However, this was not countered with the knowledge needed to navigate that risk, leaving H9 unprepared and uncertain of what the limits of their power and autonomy are in the context of restraint.

H9 went on to contemplate the drivers of their concerns about making restraint decisions.

*[The fear] comes from others*,* comes from the stories that you hear from people having to deal with management. […] That someone might sit at the top and remove all context from something. And when it comes to restraint*,* I feel it’s all context. – H9*

This extract draws attention to how colleagues’ negative experiences of ‘dealing with management*’* contribute to a sense of fear. H9 is fearful that the context critical to how a paramedic makes a restraint decision in the moment will be forgotten or ignored by those with the power to characterise decisions as right or wrong post-hoc. Specifically, there is a fear of judgement here, centred around a concern that a decision will be misjudged, due to a lack of consideration or dismissal of critical contextual information.

H5 went on to describe their broader experience of decision-making in the early days of their career, and how others’ perceptions of their practice influenced her.

*The perception from other people […] is*,* the most crippling thing to self-confidence […]. It’s the obsession of how your peers or the doctors or the nurses or the police or the family member or the public or the bus driver or whoever it is […] I think that’s the biggest influence. – H5*

H5 illuminates how the impact of others’ perceptions can influence paramedic decision-making. They highlight how their self-confidence has been influenced by the judgement of others, with negative reactions from professionals and members of the public alike causing ‘crippling’ damage to their self-confidence. Here, it is not only ‘doing the right thing’ that is important. It is also important to be perceived to do the right thing by observers.

### Theme 2: Navigating complexity without adequate formal subject-specific training

H2 drew comparisons between ABD and other high acuity presentations which are covered in greater detail in paramedic training, using cardiac arrest management as an example.

*I think if you compared cardiac arrest and ABD you would say that the cardiac arrest is relatively straightforward*,* we shock or we don’t*,* we do CPR [cardio-pulmonary resuscitation] or we don’t*,* […] whereas I think we don’t see many ABDs and when we do […] there are a lot more dynamics that you have to deal with. - H2*

H2 sheds light on the complexity of decisions in ABD cases, suggesting that they are not ‘straightforward’ or algorithmic decisions, such as those made in cardiac arrest management, which are allocated more attention in training. The implication here is that there is a mismatch between training needs and the paramedic curriculum, with a lack of formal training leaving paramedics with, in the words of H11, ‘*no idea what do to.’*.

In the next extract, H7 described attending their first case of ABD, not recognising it as ABD and underestimating the seriousness of the incident.

*I was really out of my depth […] I had absolutely no idea of any of this stuff*,* only that I just had this chance encounter with a really good paramedic […]. Otherwise*,* I probably would have just gone home*,* thought that was that was a tough job and that would have been the end of it. – H7*

Here we see how without adequate formal training paramedics are dependent on other’s professional expertise. In managing this ABD patient, H7 felt ‘out of their depth’, having had little formal education on ABD. H7’s education on ABD was accidental, part of a chance encounter, rather than planned formal learning. This lack of formal and structured education brings inherent uncertainty to clinical decision-making in ABD incidents.

It is not just confidence in managing patients and relations with other paramedics that was an issue. Below, H4 comments on the need to manage relationships with the police.

*Many of [the newer people] don’t have the same exposure to those sorts of jobs*,* so they don’t always have confidence in making decisions about when to support the police and when to*,* perhaps*,* correct what they’re doing. – H4*

Two things are evident here. Firstly, H4 links lack of experience with the capability to challenge the police if there are concerns with their manner of restraint. A lack of training in ABD is not the only issue, exposure to ABD cases and building up experience of managing them is also relevant. Secondly, it is necessary to work in conjunction with the police when dealing with an ABD incident. The development of skills in managing relationships with the police is similarly experiential and dependent on exposure to relevant contexts, as is the development of sufficient confidence to challenge the police in their decision-making and advocate for patients.

In the next example, H1, an overseas-trained paramedic expresses concern about their UK conversion training.

*I think the training around the legal aspect is very poor*,* [paramedics are] not trained well in it. It’s vaguely explained by people who have grown up in the system that also don’t know exactly how to apply it*,* and aren’t confident in it. – H1*

In this extract, H1 suggests that there is a blind-leading-the-blind situation with educators who are uncertain of the rules themselves passing that uncertainty onto others. This raises two issues. Firstly, paramedics may know what they should do clinically speaking but be uncertain whether they have the legal authority to act. Secondly, paramedics enter practice with an inherent ‘vagueness*’* about making medico-legal decisions about ABD.

In the next example, H10, an experienced female paramedic, reflects on their training specifically about restraint.

*It was like it was missing a chapter. Or*,* couple of chapters to be honest. […] We’re not taught to restrain*,* and it’s more de-escalation and that sort of thing. […] there are gonna be some situations where you’ve got no choice but you have to restrain someone. – H10*

Here, H10 draws attention to a perceived gap between what paramedics are taught and what they need to know to be able to manage these patients safely. Firstly, we see that whilst paramedic education deals with some important issues, such as de-escalation techniques, it omits ‘real-world’ actions should those techniques prove unsuccessful. As a result, paramedics go into practice unprepared to navigate the gap between their scope of practice and the tasks they are actually faced with. Secondly, there is a lack of clarity between paramedics and their employers about taking potentially controversial action in the context of ABD incidents.

### Theme 3: nature of professional roles and relationships at incident scenes

In the first example for this theme, H4 speaks of difficulties in influencing police in changing their restraint practices.

*When the police are very confident in what they’re doing*,* then it can be quite difficult to get them to behave otherwise*,* […]. To summon the authority to tell them to do otherwise. – H4*

H4 sheds light on challenges in influencing police officers. Firstly, the police are a dominant presence in these scenarios and that it is for the paramedics to influence them, rather than the other way around. Secondly, H4’s reference to police confidence hints to an idea that the decisions paramedics may seek to influence are situated within the responsibilities of the police rather than paramedics themselves. Thirdly, the police are perceived to have a high level of confidence in relation to their decisions and actions. This gives rise to a need for paramedics to draw on their own confidence in challenging restraint practice where needed. We see here that there is a tension between decision-making in the context of physical restraint and the act of restraint itself, alongside a lack of clarity in roles and responsibilities at ABD scenes.

In the next example H5 describes ABD scenes where multiple police are in attendance.

*They’ve turned out on [blue] lights. There’s a lot of them*,* and they all just came in and started YELLING*,* trying to pin this patient down and then he’s fighting back*,* and I just had to ask all of them to leave. […] They came in with so much energy […] it was just too much for what we needed. – H5*

Three issues are raised here. Firstly, the attendance of police in large numbers may have an inflammatory and in turn escalatory effect. Secondly, these large numbers may overwhelm or dominate ambulance clinicians present in fewer numbers, and thirdly, the ‘*energy*’ responders bring to ABD scenarios may be contributory to whether situations escalate or are de-escalated. In this case the entrance of a highly stimulated group seems to have escalated this case. We see here that the behaviour of a group of individuals can influence the relationships between professionals at incident scenes and have a cumulative escalatory effect.

In the following example, H11, a newly qualified paramedic, talks about being concerned about police restraint in the context of being on scene with more senior colleagues.

*So*,* if you think*,* oh I’m just an NQP and the Band 6 is in the room*,* and they haven’t said anything*,* and these officers have been officers for years. So*,* there’s a bit of that as well*,* a bit of self-doubt. – H11*

H11 sheds further light here on self-perception and how this affects decision-making. Firstly, authority gradients and the effect of others perceived to be more knowledgeable taking no action is that less experienced staff presume and perhaps learn that no action is required in the given scenario. Secondly, the reinforcement of perceived self-doubt. H11 initially believes some action to reduce restraint is required, however concludes otherwise following the inaction of those she looks to for guidance.

In the final extract, H5 speaks of self-doubt in their early career and the impact of being aware of other professionals’ perceptions of them:

*… being so aware of everyone’s perception […] [other ambulance clinicians’] eye rolls because you’re only this far into the job and [they] think we should be doing this or that*,* then the eye roll of the nurse at the hospital and the doctor’s [eye roll] and the this or the that…. – H5*

Here, we see there is a relationship between the self-perception of paramedics and how they approach decision-making in practice. Firstly, there is a sensitivity here to being judged by others on one’s performance as a paramedic. Secondly, there is a sense that self-perception is influenced by the perceptions of both peers and members of other professions. There is an important impact here of signals of judgement such as ‘*eye rolls*’ on the development of confidence in clinical practice.

## Discussion

Our findings illuminate the factors which influence paramedics when making restraint decisions in three interrelated themes. Our results support other studies which have highlighted ambulance paramedics’ experiences of fear and vulnerability in relation to decision-making in a range of contexts including resuscitation and discharge at scene [30, 34–39, 41]. ABD incidents are complex events, and paramedics operate within a context of fear arising from being in an inherently dangerous and unpredictable situation, as well as concerns about patients deteriorating because of underlying pathology or the effects of physical restraint. As seen in other research, here paramedics were also fearful of adverse medico-legal or disciplinary consequences if they did intervene, with an inherent uncertainty about the limits of their autonomy linked to a mismatch between policy and practice [32, 36]. Paramedics were concerned about how their actions would be perceived by their managers and appeared to mistrust them, fearing that they might lose their jobs because of providing restraint, echoing research that finds paramedics perceive a culture of discipline and blame within UK ambulance services and operate in fear of repercussions [58].

Overlapping with and contributing to these fears was a sense that paramedics felt poorly prepared to operate in this high-risk context. Whilst greater training was available to more recently qualified paramedics, there was a lack of formal education and training identified in three key areas: medicolegal duties and rights, ABD as a clinical entity, and physical restraint itself. This lack of formal education and training, whether real or perceived, contributes to inherent complexity and an ambiguous decision-making context. This is mirrored in a recent study of Norwegian ambulance staff’s experience of physical force coercion [24], as well as studies on non-conveyance decision-making [29] studies relating to paramedics in the context of mental health emergencies and self-harm [32] and managing older fallers [39]. Paramedics relied on experiential learning, echoing the experience of nurses in relation to seclusion and restraint groups [25]. Exposure to discretionary education on ABD and restraint was variable, perhaps increasing the disparity in support available to junior staff, including those in their preceptorship period. A lack of consistency in education also risks a lack of common understanding in recognising and managing ABD, raising the potential for variation in interpretation of guidance and for conflicting advice.

There was variation in which country participants undertook their qualifying training, with five of the 13 participants qualifying in Australasia and eight qualifying in the UK. Both groups of paramedics reported issues with their experience of UK education regarding medicolegal matters, the restriction of liberty and assessment of mental capacity. Participants revealed different perspectives on the restriction of liberty, which may reflect the different legal and regulatory contexts of UK and Australasian ambulance services. The curricula for pre-registration paramedic education is likely to differ between both regions in relation to this and other areas. The requirement to transition from one jurisdiction to another for Australasian trained participants suggests there are implications for paramedics trained in other systems moving to the UK to practice and may indicate a need for further research on the implications of this.

Participants obtained some of their information on an ad-hoc basis and learnt from others, sometimes as part of a chance encounter. This risked gaps in knowledge and adoption of poor, or poorly evidenced, practices, and as in other professional groups [25], the repetition of errors. Some participants reported learning from colleagues perceived to have special knowledge of ABD, such as APs. Individuals who are considered to be reliable sources of information hold considerable influence over others [59]. However, being seen as a reliable source of information does not necessarily mean the information one holds is accurate. Given the apparent gap in pre-registration education on ABD and restraint, some non-specialists might be seen as influential but lack the knowledge needed to accurately guide others. This may include those in formal supervisory or managerial roles, as well as informal leaders.

These issues were further complicated by the need to manage relationships with other professionals, usually the police, at ABD incident scenes and how paramedics characterised their roles as healthcare professionals. We found that key influences on decision-making in ABD arose from the social and professional relationships between responders. There was a sense that a lack of empowerment complicated participants’ abilities to fulfil their duty of care. Participants actively situated incidents as healthcare encounters, this context was key to locating ABD incidents in the span of control of paramedics within power gradients at the scene.

Police officers were perceived to hold high social authority and expertise in managing agitation and restraint, and the experience levels of paramedics were relevant to how confident they felt in challenging police officers’ decisions. Participants described ABD patients being restrained by multiple police officers, and there sometimes being resistance to requests for a reduction in physical restraint or other strategies for de-escalation. We identified that paramedics take active steps to situate ABD incidents as healthcare, rather than policing encounters in response to this issue. rather than healthcare professionals.

These results echo those of a related study sampling APs, who reported some similar concerns particularly in relation to the use of chemical restraint and the deteriorating patient. Sedation is available to specific cohorts of advanced paramedics, however is used comparatively rarely in the UK [60]. Unlike the participants in the present study, APs were more concerned about the aetiology of ABD and how this might impact decisions to administer chemical restraint, particularly in relation to drug and alcohol intoxication which may increase the risk of deterioration following sedation [48].

This study sought to shed light on how clinical decisions are made in relation a particular group of patients who are commonly described as presenting with ABD. As highlighted earlier, use of this terminology is controversial, as is the term excited delirium where used. Terms such as excited delirium (often used interchangeably with ABD) are disproportionately applied to black men in police custody [61]. Some suggest that this label has the potential to cause harm to patients, with Polling et al. [62], finding that black people were twice as likely as white people to have a reference to ABD in the records of their acute mental health assessments, concluding that by way of the link between ‘ABD’ and risk of collapse that this may contribute to racial inequalities in the use of coercion during a mental health crisis. Elijah McClain died in 2019 in the USA. He was thought to be suffering from excited delirium and was sedated by paramedics with a large dose of ketamine. He suffered a cardiac arrest and died, with both paramedics subsequently being convicted of criminally negligent homicide [63]. The following year George Floyd died following physical restraint by police in the USA. Reports suggested he was suffering from excited delirium [64]. A police officer, Derek Chauvin, was subsequently convicted of Mr. Floyd’s murder. These cases are tragic illustrations of why some seek abandonment of these terms, in that they are used to deflect attention from restraint as a cause of death and that its use serves to invite excess restraint in the first place with a growing movement towards abandoning the term excited delirium [10, 12–14, 65, 66] and to a lesser degree the term ABD [11].

Whilst this study did not seek to answer questions in relation to how this patient group should be classified, our analysis contributes to this debate. Issues in relation to the ethnicity of patients were specifically raised by one participant as adding to their concerns in relation to the potential for excessive restraint, the perceptions of onlookers and the resultant risk of escalating tensions between police and bystanders. Added to this, there was a sense of ambiguity, fear and threat in relation to managing ABD patients, and we know that prejudice towards a group [67] and perceived threat [68] lead to greater reliance on bias when making decisions. Regardless of the outcome of the debate in relation to terminology, the findings of this study shed light on how emergency medical services and the police may better respond to this patient group.

Our findings also contribute to the international evidence base which suggests that ambulance paramedics experience vulnerability and fear concerning high-risk decision-making. This is an important patient safety issue which also risks the health and well-being of paramedics. More action is urgently needed to improve psychological safety, or the “…*belief that one can speak up without risk of punishment or humiliation.”* [69] to address this.

This study also calls attention to a gap in formal education and training in respect of ABD and restraint decision-making more broadly. A greater emphasis on preparing paramedics to differentiate ABD from other types of agitation in formal education, and to navigate restraint decisions is needed. This is particularly important in relation to medico-legal matters and patient safety and should take explicit account of the needs of learners recruited internationally who may have trained in different legal and regulatory contexts. Education which addresses the role and responsibilities of the police and others involved in the care of ABD patients, and how to interpret and manage the complex social dynamics of incidents is also needed, including the management of leadership dynamics.

Our analysis also highlights the role of context and social relations in shaping how decisions are made in paramedic practice. In preparing for clinical practice, issues around confidence and primacy of care represent important opportunities for development.

### Reflexivity

The three authors involved in the analysis were a woman consultant paramedic interested in medical ethics, professional duty and patient safety (JL); a man consultant paramedic with extensive experience in developing and providing advanced paramedic critical care services (TE); and a senior woman social psychologist who researches contextually situated social interactions and decision-making (LB).

Based on her personal experience of training, the first author (JL) initially brought assumptions that issues of individual decision-making would be emphasised in the data and the centrality of group processes and social influence were not anticipated. Furthermore, that participants might be concerned about repercussions was expected, although engagement with the data identified that this issue was much broader than initially imagined, particularly in relation to formal and informal organisational culture. In relation to the educational concerns raised by participants, it was initially assumed that issues would centre around formal curricula, however on reflection more informal influences, such as warnings, were identified as important.

JL and TE both brought experience of practicing as paramedics at the study site for many years, and TE brought extensive experience of managing ABD incidents and providing sedation. LB brought a deep social psychological lens on group processes to the analytic discussions. The authors engaged in frequent conversations during analysis to discuss interpretations of the data and challenge analytic assumptions.

### Limitations

This is a relatively small study of thirteen participants. All participants were white therefore the perspectives of global majority participants were not available. Given the racial inequalities in relation to ABD which have been highlighted this may mean that important factors in relation to diagnostic labelling and the overuse of restraint may have been overlooked. Efforts were made to broaden the ethnicity of the sample by advertising via the study site’s diversity staff network, however these were unsuccessful.

This study took place in a single UK ambulance service setting. Participants were all self-selected volunteers and may have a personal interest in ABD. This may indicate that our participants may have had a greater level of interest in ABD in comparison to the general paramedic population. Our study only included paramedics, and therefore the perspectives of other key agents, such the police, were not available.

Although interviews were in-depth and produced a rich data corpus it is recognised that additional observational techniques would have contributed further robustness to the study’s findings.

## Conclusion

This study is the first to research the influences on paramedic decision-making in the context of restraining patients presenting with ABD and brings new and deep understanding of the restraint decisions made by paramedics in this context. This is particularly important given recent concerns relating to deaths in the context of restraint [70, 71] and the findings have important implications for future practice.

Increasing education and training concerning the pathophysiology of agitation and its causes, alongside the development of in-depth knowledge of relevant legal frameworks present as opportunities to reduce the uncertainty and ambiguity paramedics experience in this area of practice.

In addition to addressing clinical and medico-legal knowledge issues, our findings also suggest that further attention to the social environment in which agitated patients present and paramedics work is warranted. Education and development activities which help paramedics to navigate a context in which they must negotiate with other professional groups to establish leadership and primacy of care presents an important improvement opportunity. Our findings suggest that a much greater emphasis on the social interactions which form part of the context in which restraint decisions are made is warranted when designing educational curricula and practice guidance.

## Supplementary Information

Below is the link to the electronic supplementary material.

Supplementary Material 1

Supplementary Material 2

## Data Availability

Anonymised transcripts are available via the University of Bath Research Data Archive. The data is available to researchers on application, rather than on an open access basis. This is to protect the privacy of participants and this requirement forms part of the ethical approval for this study. The reference and DOI for the data will be as follows: Lindridge, J., Edwards, T., Blackwood, L., in press. Dataset for: Decision-making and acute behavioural disturbance (ABD): A qualitative thematic analysis of perspectives on decision-making by UK ambulance paramedics. Bath: University of Bath Research Data Archive. https://researchdata.bath.ac.uk/id/eprint/1486. The DOI will be activated on publication of the manuscript.

## Abbreviations

ABD: Acute behavioural disturbance
AP: Advanced Paramedic
CPR: Cardio-pulmonary resuscitation
EMT: Emergency Medical Technician
FHEQ: Framework for higher educational qualifications
NQP: Newly Qualified Paramedic
PGD: Patient group direction
RTA: Reflexive thematic analysis
SI: Serious incident
UK: United Kingdom
