# Supplementary material for: Decision-making and acute behavioural disturbance (ABD): a qualitative thematic analysis of perspectives on decision-making by UK ambulance paramedics
Source: BMC Emerg Med. 2025 Jul 26;25:135. doi: 10.1186/s12873-025-01297-7 (PMC12297720; doi:10.1186/s12873-025-01297-7)
Supplement: Supplementary file 1 — Supplementary Material 1 [file 12873_2025_1297_MOESM1_ESM.pdf]

SEMI STRUCTURED INTERVIEW GUIDE

A qualitative exploration of restraint decisions made by paramedics and advanced paramedics when managing patients presenting with acute behavioural disturbance (ABD) in the pre-hospital setting.

**Welcome & introductions**<br><br>**Role**<br>**Work**<br>**Location**	Outline of the purpose of the research. I'm interested in paramedic's experiences of dealing with ABD particularly around restraint, interested in paramedic's stories.<br><br>Informed consent, withdrawal and confidentiality of data. Clarity on confidentiality and participant safety.<br><br>Insider researcher, but I may ask questions to clarify things in areas which you would expect me to understand – this is for the purposes of the transcripts.<br><br>Feel free to have your camera on or off. Reiterate the safety of the conversation and right to withdraw consent without giving a reason. No obligation on continuing.<br><br>Any questions? **Start recording and transcription support.**
**Introductory questions**	(This is not a test!) What do you understand ABD to mean?<br><br>What does restraint mean to you as a concept?<br><br>**Can you tell me a story about a time when you had to decide whether to restrain a person with ABD or not?**
**Variations, probes and extensions**	How did things unfold?<br>Who was providing the restraint? Who determined it was necessary?<br><br>Who else was there? Who else did you speak to? (**interactions with other paramedics, police, other professionals**)<br><br>Can you tell me a little more about those interactions…?<br><br>What are the roles of the professionals in these cases?<br>What are the expectation of paramedics/ advanced paramedics in these scenarios? (**employer, professional, patient…**)<br><br>How do you feel about managing cases of ABD?<br>Can you tell me more about your training and education in this area of practice?<br>How do you learn for these scenarios?<br><br>Thinking back to that experience ...<br>Can we go back and talk more about…<br><br>Can you tell me about any cases where you have not provided restraint?<br>Can you give me an example of a time when you have/ have not decided that restraint was necessary?<br><br>What was it about the experience that stood out?<br><br>Why did you to manage the patient in that way?<br><br>All of all of things we have discussed, what is the most important to you?

Version 0.3

	Do you have any examples of restraint decision-making outside of ABD?<br><br>Do you have any more stories?<br><br>Is there anything we haven't discussed which you like to say?
**Debrief**	Debrief and thank participant for their participation.

Version 0.3
