# Supplementary material for: Decision-making and acute behavioural disturbance (ABD): a qualitative thematic analysis of perspectives on decision-making by UK ambulance paramedics
Source: BMC Emerg Med. 2025 Jul 26;25:135. doi: 10.1186/s12873-025-01297-7 (PMC12297720; doi:10.1186/s12873-025-01297-7)
Supplement: Supplementary file 2 — Supplementary Material 2 [file 12873_2025_1297_MOESM2_ESM.pdf]

FOCUS GROUP GUIDE

A qualitative exploration of restraint decisions made by paramedics and advanced paramedics when managing patients presenting with acute behavioural disturbance (ABD) in the pre-hospital setting.

**Welcome & introductions**	Outline of the purpose of the research.<br><br>Informed consent, withdrawal and confidentiality of data. Declarations for members of the University of Bath, who should undertake not to record the session.<br><br>Guidelines for participation: one person speaking at any given time, respectful listening and accept others may not agree, confidentiality and process for escalating concerns.<br><br>Technical instructions if required, signpost to 'raise hand' function and chat box which participants can use if desired.
**Introductory question**	I'm interested in hearing about your experiences of managing ABD patients.<br><br>**What's the first thing which comes to mind when thinking about restraining ABD patients?**<br><br>Would anyone like to start with a case which stands out?
**Variations, probes and extensions**	How did things unfold?<br><br>What happens when you ... (interactions with other paramedics, police, other professionals)<br><br>How do you feel about managing cases of ABD?<br><br>Thinking back to that experience ...<br><br>What do others think?<br><br>Can anyone tell me about any cases where you have not provided restraint?<br><br>Could you please elaborate on ...<br><br>Can you give me an example of a time when you have/ have not decided that restraint was necessary?<br><br>Can you tell me more about ...<br><br>What was it about the experience that stood out?<br><br>Does that resonate with others?<br><br>What influenced you to manage the patient in that way?<br><br>All of all of things we have discussed, what is the most important to you?

Version 0.3

	Is there anything we haven't discussed which you like to say?<br><br>• How do you feel about those risks?<br>• Can you tell me more about the patient?<br>• Why were they acting like that?<br>• What sort of support would you expect to get?<br>• What sort of things were you weighing up?<br>• What made it difficult/ easy?<br>• How do you feel about restraining patients?<br>• Is it different when using medicines, as in the APPs sedating?<br>• What do you think your role in restraining ABD is/ should be?<br>• What have you heard?
**Closing**	Does anyone have any other thoughts or view that they would like to share?<br><br>Was there any particular reason you wanted to participate in this focus group?
**Debrief**	Debrief and thank participants for their participation.

Version 0.3
